# Specific metabolites drive the deterministic assembly of diseased rhizosphere microbiome through weakening microbial degradation of autotoxin

**DOI:** 10.1186/s40168-022-01375-z

**Published:** 2022-10-21

**Authors:** Tao Wen, Penghao Xie, C. Ryan Penton, Lauren Hale, Linda S. Thomashow, Shengdie Yang, Zhexu Ding, Yaqi Su, Jun Yuan, Qirong Shen

**Affiliations:** 1 The Key Laboratory of Plant ImmunityJiangsu Provincial Key Lab for Organic Solid Waste UtilizationJiangsu Collaborative Innovation Center for Solid Organic Waste Resource Utilization, National Engineering Research Center for Organic-based Fertilizers, Nanjing Agricultural University, Nanjing, 210095 China; 2 Center for Fundamental and Applied Microbiomics, Biodesign Institute, Arizona State University, Tempe, AZ 85287 USA; 3 Faculty of Science and Mathematics, College of Integrative Sciences and Arts, Arizona State University, Mesa, AZ USA; 4 Agricultural Research Service, USDA, San Joaquin Valley Agricultural Sciences Center, Parlier, CA 93648 USA; 5 Agricultural Research Service, US Department of Agriculture, Wheat Health, Genetics and Quality Research Unit, Pullman, WA 99164 USA

**Keywords:** Microbial community assembly, Phylogenetic pattern, Rhizosphere metabolomics, Fusarium wilt disease, Integration analysis metadata

## Abstract

**Background:**

Process and function that underlie the assembly of a rhizosphere microbial community may be strongly linked to the maintenance of plant health. However, their assembly processes and functional changes in the deterioration of soilborne disease remain unclear. Here, we investigated features of rhizosphere microbiomes related to Fusarium wilt disease and assessed their assembly by comparison pair of diseased/healthy sequencing data. The untargeted metabolomics was employed to explore potential community assembly drivers, and shotgun metagenome sequencing was used to reveal the mechanisms of metabolite-mediated process after soil conditioning.

**Results:**

Results showed the deterministic assembly process associated with diseased rhizosphere microbiomes, and this process was significantly correlated to five metabolites (tocopherol acetate, citrulline, galactitol, octadecylglycerol, and behenic acid). Application of the metabolites resulted in a deterministic assembly of microbiome with the high morbidity of watermelon. Furthermore, metabolite conditioning was found to weaken the function of autotoxin degradation undertaken by specific bacterial group (*Bradyrhizobium*, *Streptomyces*, *Variovorax*, *Pseudomonas*, and *Sphingomonas*) while promoting the metabolism of small-molecule sugars and acids initiated from another bacterial group (*Anaeromyxobacter*, *Bdellovibrio*, *Conexibacter*, *Flavobacterium*, and *Gemmatimonas*).

Video Abstract

**Conclusion:**

These findings strongly suggest that shifts in a metabolite-mediated microbial community assembly process underpin the deterministic establishment of soilborne Fusarium wilt disease and reveal avenues for future research focusing on ameliorating crop loss due to this pathogen.

**Supplementary Information:**

The online version contains supplementary material available at 10.1186/s40168-022-01375-z.

## Background

While the rhizosphere microbial community plays an important role in both plant growth and health [1], alterations in the rhizosphere microbiome that negatively impact diversity and/or composition can result in soilborne disease with concomitant negative effects on plant productivity [2–5]. Among the soilborne plant diseases, Fusarium wilt, caused by the fungal pathogen *Fusarium oxysporum*, is highly destructive and has a broad host range in agricultural production systems [6, 7]. Infection is initiated in root tips followed by migration into immature xylems, ultimately resulting in plant symptoms such as root rot, vascular wilt, and damping off [8]. Changes in the composition of the rhizosphere microbial community due to the presence of fusarium wilt disease have been documented previously [9–11], with attempts to identify the underlying mechanisms that drive the emergence of the disease [10]. However, due to the complexities of the soil ecosystem and interactions between plant and soil type, legacy effects, climate, pH, and other factors, literatures on the relationship between the rhizosphere microbiome and fusarium wilt disease are incongruent. Nevertheless, a metadata analysis approach undertaken by integrating publicly available sequencing data can be used to address these discrepancies and identify common responses across systems and plant types [12]. Our previous study examined fusarium wilt in relation to the bacterial and fungal communities of healthy and diseased soils by using a machine-learning approach. Results showed that the communities were significantly different in compositions and further identified 45 bacterial and 40 fungal OTUs that predicted the health status of the soil with high accuracy [13], while little is known about how microbial community assembly processes in diseased rhizosphere microbiome.

A myriad of abiotic and biotic factors can impact the assembly of a rhizosphere microbial community. Root exudates, which act as both a source of nutrients and signaling molecules, are expected to play a significant role within the rhizosphere environment [14, 15]. However, root exudates can impart both beneficial and harmful impacts on plant-microbe interactions. For example, some metabolites have the capacity to recruit beneficial microbes as a defense against pathogens, while others negatively impact the composition of the rhizosphere microbial community [15, 16]. This negative impact can lead to a pathogen-dominated “diseased” microbiome, especially under long-term continuous cropping conditions [15]. Among the metabolites exuded from the root, cinnamic acid specifically has been reported to promote the incidence of Fusarium wilt in *Cucumis* by increasing pathogen abundance [17, 18]. Though metabolites were recognized to be important for rhizosphere microbial community assembly, the composition of rhizosphere metabolites was affected by various factors such as host plant species, soil types and growth status of host plant [19]. However, metabolites that regulate microbial community assembly in relation to a healthy host plant are yet to be identified.

In order to address these questions, we merged sequencing data originating from Fusarium wilt diseased and healthy plant rhizospheres and then evaluated the assembly process of the microbial communities by calculating the β-nearest taxon index (βNTI). In addition, untargeted metabolomics was used to identify specific metabolites that influence rhizosphere microbiome assembly. Identified “key” metabolites were then used to induce a microbial community that reflects a “diseased” state. Overall, we aimed to address the following: (1) whether the phylogenetic patterns of the microbial community are different between diseased (Fusarium wilt) and healthy rhizosphere soils and (2) what processes do rhizosphere metabolites drive the phylogeny of a diseased rhizosphere microbial community. We hypothesized that certain metabolites in root exudates could drive the assembly process of rhizosphere microbial communities, resulting in a “susceptible” microbiome under pathogen attack.

## Results

### The deterministic assembly process of microbial community was found in diseased rhizosphere soil

**Fig. 1:**
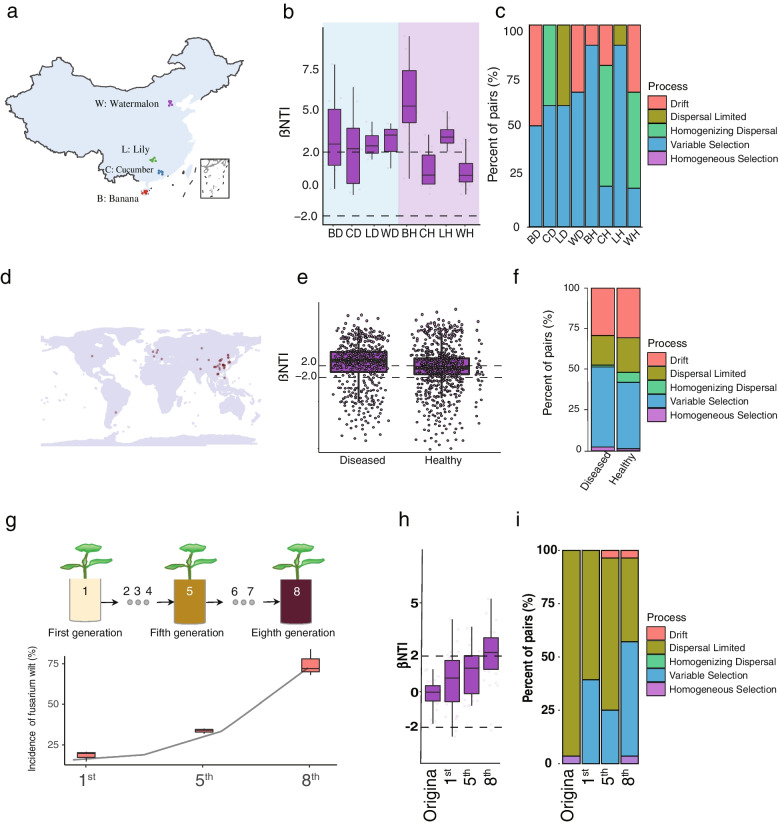
**a** Schematic picture for the location of rhizosphere soils sampling. **b** Contributions of deterministic and stochastic processes in community assembly within collected rhizosphere soil samples. βNTI calculation of phylogenetic turnover among diseased and healthy samples indicates that variable selection was more consistent in diseased soils. **c** The relative influence of each community assembly process among diseased and healthy samples was defined by the percentage of site pairs governed by each process. BD, diseased banana; BH, healthy banana (from Hainan); CD, diseased cucumber; CH, healthy cucumber (from Guangdong); WD, diseased watermelon; WH, healthy watermelon (from Beijing); LD, diseased lily; LH, healthy lily (from Hunan). **e** Contributions of deterministic and stochastic processes on community assembly within diseased and healthy soils of collected metadata. βNTI calculations of phylogenetic turnover between diseased and healthy soils indicate that variable selection has greater effects on disease than health. **f** The relative influence of each community assembly process between diseased and healthy soils as defined by the percentage of site pairs governed by each process. **g** Disease incidence of the first, fifth, and eighth generation. **h** Contributions of deterministic and stochastic processes on community assembly within pot experiment. **i** The relative influence of each community assembly process of rhizosphere soil samples from pot experiment

We performed three experiments to evaluate the assembly process of diseased rhizosphere microbial communities. Firstly, four crops of rhizosphere soil samples, both diseased and healthy, were collected to assess the assembly process (Fig. 1a). The β-nearest taxon index (βNTI) between sample pairs was calculated. Consistent variable selection was observed across all the diseased rhizosphere bacterial communities (βNTI > 2), while various directions were shown within healthy samples (2 pairs of βNTI > 2, 2 pairs of |βNTI| < 2) (Fig. 1 b–c). Furthermore, a stochastic process (|βNTI| < 2) dominated the phylogenetic turnover in the healthy rhizosphere bacterial communities (Fig. 1b). Secondly, a total of 1722 samples from 45 individual bacterial studies (Supplementary Fig. 1; Supplementary Table 2) were collected to further validate assembly process, and results showed a variable selection of diseased rhizosphere microbiome (βNTI > 2). The stochastic process (|βNTI| < 2) dominated the phylogenetic turnover in the healthy rhizosphere microbiome (Fig. 1 d–f), and homogenizing dispersal (RCbray < −0.95) was found to be dominant among the stochastic processes (Fig. 1f).

To further determine the variable selection dominated by the assembly process of the bacterial community, we conducted a pot experiment for simulation of the formation process of disease conducted soil, and rhizosphere was sampled from each generation of plant for 16S rRNA gene sequencing. With the increasing generations of continuous cropping, the level of fusarium wilt gradually increased (Fig. 1 g) from 1st generation to 8th generation, and the value of βNTI was also gradually increased to βNTI > 2 at 8th generation (Fig. 1 h–i).

### Excavation of special metabolites driving rhizosphere microbial community assembly process

Four pairs of rhizosphere soil samples from Hainan, Guangdong, Beijing, and Jiangsu province were analyzed by GC-TOF-MS, resulting in a total of 798 chromatographic peaks with 265 identified metabolites across all samples. This included 45 amino acids and amides, 23 alcohols, 21 long-chain carbon organic acids, 27 short-chain carbon organic acids, 5 nucleotides, 36 sugars, 7 sugar acids, 4 sugar alcohols, 8 esters, and 89 others (Supplementary Table 5). Principal coordinates analysis (PCoA), based on Bray-Curtis distances, illustrated that the rhizosphere metabolites were dissimilar among all samples (*p* = 0.001, PERMANOVA by Adonis) (Supplementary Fig. 2), and pairwise comparisons with samples from each site confirmed significant differences between the diseased and healthy (Supplementary Table 6).

**Fig. 2:**
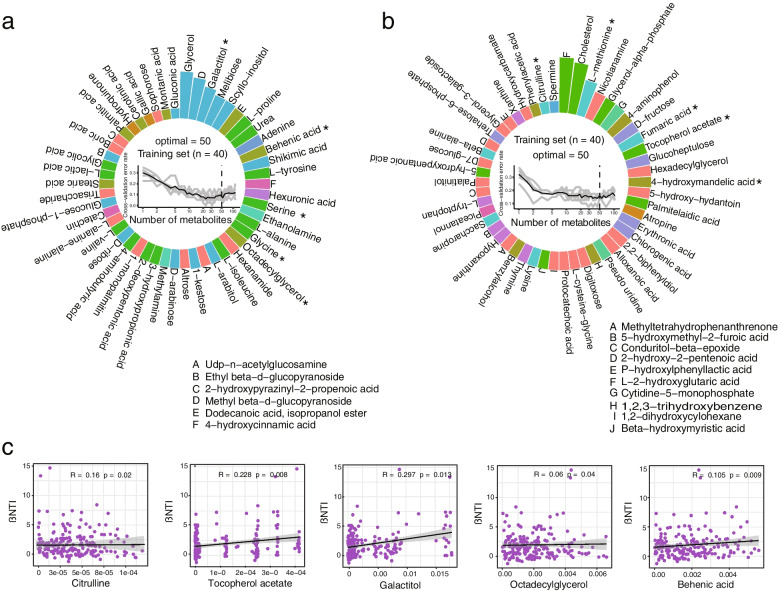
**a** Random forest models built with the most abundant rhizosphere metabolites and the top 50 most important metabolites. The five featured metabolites are denoted with asterisks. **b** Random forest models constructed with the lower abundant rhizosphere metabolites and the top 50 most important metabolites. The five featured metabolites are denoted with asterisks. **c** Scatter plots illustrating the correlation between βNTI and the relative abundance of the five featured metabolites. *p*-values were evaluated and significant correlations were determined at *p* < 0.05

A random forest model was then used as a classifier in order to distinguish the metabolites associated with the diseased and healthy rhizosphere soils. Two models were able to identify two major groups of metabolites (Fig. 2 a–b; Supplementary Figs. 3 and 4), with a total of 100 metabolites found to be the best biomarkers. By using a nonparametric test, 130 metabolites were found to exhibit significant differences (*p* < 0.05) between the diseased and healthy groups. A total of 46 metabolites were selected by combining the results of random forest and variation analysis, which accounted for the majority of the significant difference between the two groups (Supplementary Table 7). We selected the metabolites enriched in diseased groups and finally identified five metabolites (tocopherol acetate, citrulline, galactitol, octadecylglycerol, and behenic acid) which were significantly correlated with βNTI, based on a Mantel test (Fig . 2c, *p* < 0.05). These five metabolites were used for further validation of their effect on microbial community assembly and disease occurrence.

### Special metabolites drive deterministic process of diseased microbial community assembly

**Fig. 3:**
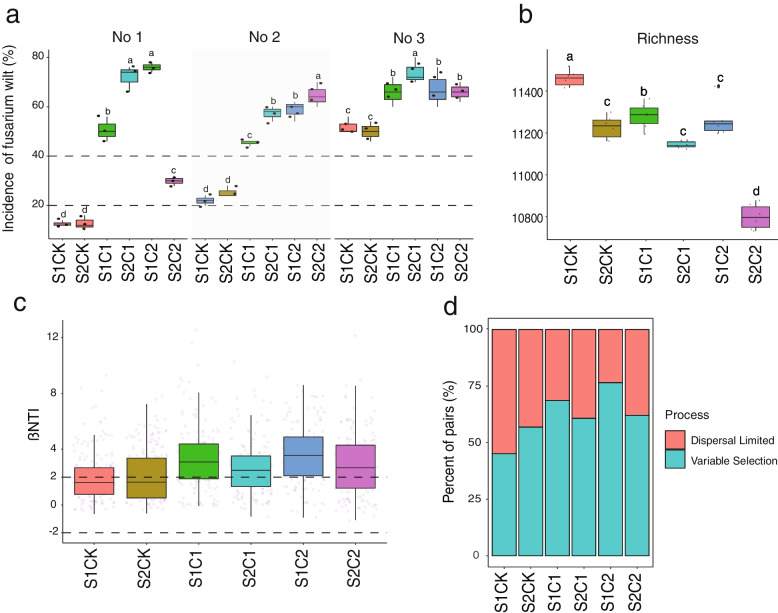
**a** Effect of the five featured metabolites on the disease incidence of fusarium wilt. NO1, NO2, and NO3 indicate three independent validation experiments. **b** Alpha diversity (richness) of soil bacterial communities after being conditioned with the featured metabolites or water control. **c** Contributions of deterministic and stochastic processes on community assembly in the sampling data. βNTI calculations of two soils conditioned with metabolites indicate that variable selection has greater effects on disease than health. **d** The relative influence of each community assembly process of two soils conditioned with metabolites as defined by the percentage of site pairs governed by each process. S1C1 means soil1 conditioned by metabolites at a concentration of 1 μM, S1C2 means soil1 conditioned by metabolites at a concentration of 100 μM, S2C1 means soil2 conditioned by metabolites at a concentration of 1 μM, and S2C2 means soil2 conditioned by metabolites at a concentration of 100 μM. Different letters mean significant difference among groups (*p* < 0.05, Wilcoxon *t*-test)

For the validation experiment, two soils were conditioned by using the five previously identified metabolites at two application concentrations (1 μM and 100 μM). After 8 weeks, cultured soils were used for a disease incidence validation experiment. Fusarium wilt incidence of watermelon was significantly higher in the treatments with soil slurries which had been conditioned with the metabolites at a concentration of 1 μM and 100 μM (Fig. 3a). Compared with the control, the incidence of fusarium wilt was increased about 25.33 (C1)–37.11% (C2) for seedlings grown in soil 1 and 26.88 (C1)—38.44% (C2) in soil 2. We then analyzed the bacterial communities of soils after conditioning with the metabolites. There were significant differences (Adonis, *p* = 0.04, *R* = 0.78, PERMANOVA) between the composition of the bacterial communities among treatments (Supplementary Fig. 5). This was accompanied by a decrease in *alpha* diversity, especially in the 100 μM treatment (Fig. 3b). Both 1 μM and 100 μM treatments (C1, C2), deterministic processes, dominated with βNTI > 2, while |βNTI| < 2 was found in the two controls (Fig. 3 c–d)

### Functional profiles and their microbial contributors of both metabolite-driven and plant-driven diseased soil

**Fig. 4:**
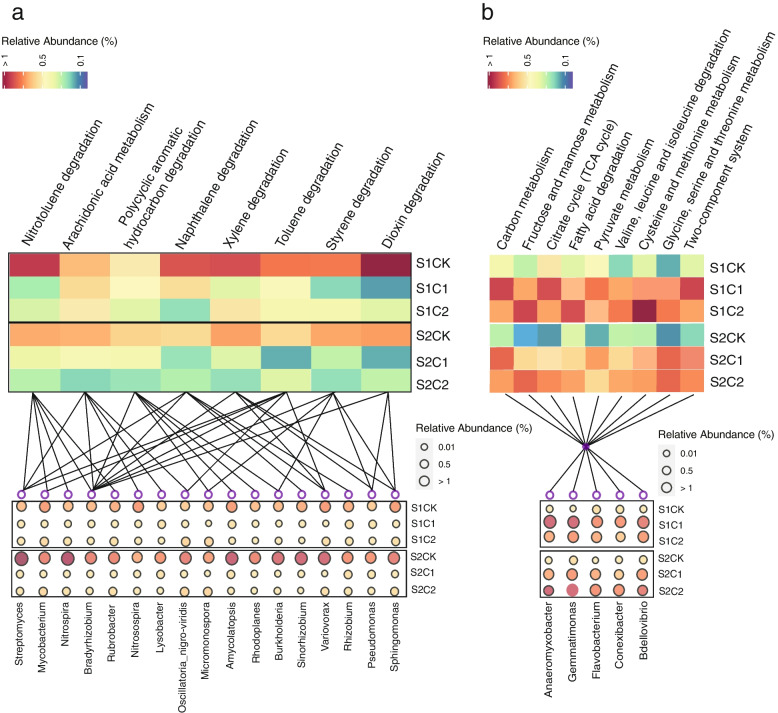
**a** Consort diagram with heatmap and bubble plot showed the depleted pathways after metabolites application. Heatmap part showed the pathways enriched in S1CK and S2CK, while bubble plot showed their contributed microbes. **b** Consort diagram with heatmap and bubble plot showed the enriched pathways after metabolites application. Heatmap part showed the pathways enriched in S1C1, S1C2, S2C1, and S2C2, while bubble plot showed their contributed microbes

**Fig. 5:**
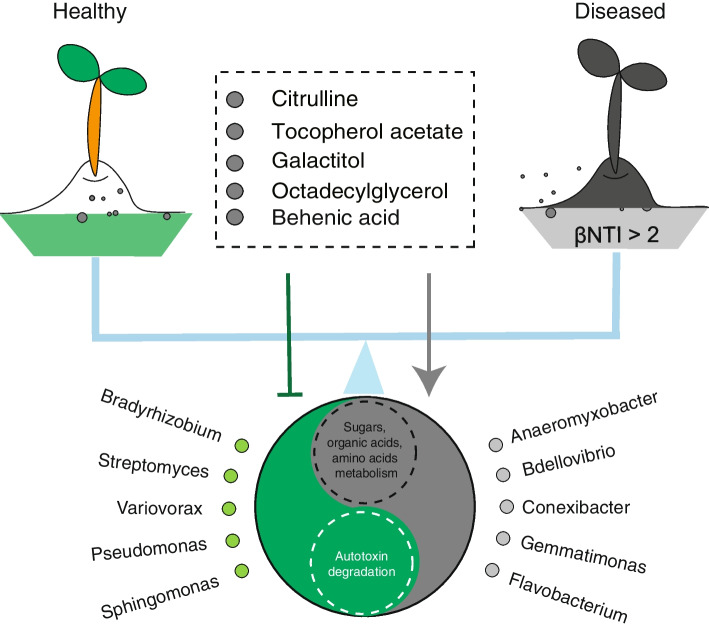
Schematic model of metabolites drives the deterministic community assembly of Fusarium wilt-diseased rhizosphere microbiome through weaken microbial degradation of autotoxin

To explore the functional variations of soil microbiome after conditioned by special metabolites, soil samples were shotgun metagenome sequenced, and 1080G raw data from 36 samples with about 30G per sample were obtained. The PCoA with Bray-Curtis distance showed a significant (MRPP: delta: 0.06; *p* = 0.001) difference among treatments (Supplementary Fig. 6). GSVA enrichment analysis was conducted between pair of two groups (S1C1 vs S1CK; S1C2 vs S1CK; S2C1 vs S2CK; S2C2 vs S2CK), and the functional pathways significantly (*p* < 0.05, two-sided unpaired limma) enriched in multiple pairs (3/4) were considered as the “important pathway”. These pathways included mainly autotoxin degradation (such as nitrotoluene degradation, arachidonic acid metabolism), the small-molecule sugars metabolism (such as fructose and mannose metabolism), organic acids metabolism (such as citrate cycle metabolism, fatty acid degradation, pyruvate metabolism), and amino acids metabolism (such as valine, leucine, and isoleucine degradation, cysteine and methionine metabolism) (Supplementary Figs. 7, 8, 9, and 10). Then, up- and depleted pathways after special metabolite application were summarized separately. The ability of autotoxin degradation (e.g., nitrotoluene degradation, arachidonic acid metabolism, polycyclic aromatic hydrocarbon degradation, naphthalene degradation, xylene degradation, toluene degradation, styrene degradation, and dioxin degradation) was significantly depleted by special metabolite application (Fig. 4a; Supplementary Figs. 7, 8, 9, and 10). Those functions were primarily mediated through a feature microbial group (FM1), including *Bradyrhizobium*, *Streptomyces*, *Variovorax*, *Pseudomonas*, and *Sphingomonas* (Fig. 4a). The metabolism pathway of small-molecule sugars (fructose and mannose metabolism) and organic acids and amino acids (citrate cycle metabolism; fatty acid degradation; pyruvate metabolism; valine, leucine, and isoleucine degradation; cysteine and methionine metabolism; glycine, serine, and threonine metabolism) was significantly enriched by metabolites application (Fig. 4b). Those functions were primarily mediated through another feature microbial group (FM2) including *Anaeromyxobacter*, *Bdellovibrio*, *Conexibacter*, *Gemmatimonas*, and *Flavobacterium* (Fig. 4b and Fig. 5).

Then, in order to further confirm the functional features of diseased rhizosphere soil, samples from the first generation (1st, recognized as health) and eighth generation (8th, recognized as disease) were also shotgun metagenome sequenced and showed a significant (MRPP: delta: 0.051; *p* = 0.028; Supplementary Fig. 11) difference. GSVA enrichment analysis confirmed the functions of polycyclic aromatic hydrocarbon degradation, and arachidonic acid metabolism was driven by FM1 and depleted, while the metabolism of small-molecule sugars, organic acids, and amino acids was driven by FM2 and enriched in 8th rhizosphere soil (Supplementary Fig. 12). Variation analysis of microbial composition showed the relative abundances of bacteria in FM1 were increased from health to disease (from 1 to 8), while the relative abundances of bacteria in FM2 showed the opposite trend (Supplementary Fig. 13). Besides functionality of autotoxin degradation and other functions (ubiquinone and other terpenoid−quinone biosynthesis, biosynthesis of vancomycin group antibiotics, and biosynthesis of enediyne antibiotics) were enriched in healthy rhizosphere microbiome (Supplementary Fig. 12).

## Discussion

In this study, we combined global bacterial high-throughput sequencing data of fusarium wilt rhizosphere-associated soil samples from multiple independent studies and crops for the identification of microbial community characteristics associated with disease. Lower bacterial community diversity was associated with disease, in concert with earlier findings [20]. Based on network analyses, a low number of connections were associated with the diseased network, reflecting less robust microbe-microbe interactions within the community. Previous studies also associated disease with lower connectivity in microbial networks. For instance, the number of network edges decreased in a fusarium wilt diseased microbial network in banana [21], and more connections were present in a network associated with healthy rhizosphere soils rather than diseased samples [22]. The presence of *Kaistobacter*, *Mesorhizobium*, *Bacillus*, *Anaeromyxobacter*, *Bdellovibrio*, *Conexibacter*, and *Flavobacterium* in the diseased samples was determined as the microbial feature that distinguished the diseased rhizosphere microbiomes. However, the majority of the top 50 most abundant microbial taxa identified through cross-validation were also more abundant in diseased rhizosphere soils than in healthy soils. This indicates that a diseased microbiome may have more uniform characteristics than that of a healthy and diverse microbiome. Diseased communities also exhibited lower variation in community composition among samples, compared to larger variations exhibited by the healthy samples. This infers a homogenization effect associated with biotic stress from fusarium wilt disease that is similar to the impact of abiotic stresses such as drought and salinity [23]. This homogenization effect served as a basis to examine the rhizosphere microbiome assembly processes under fusarium wilt disease pressure.

Four basic processes (diversification, dispersal, selection, and drift) can contribute to microbial community assembly [24] and subsequently can be used to describe the microbial assembly process under different environmental scenarios [25–27]. In this study, we explored the assembly process of the rhizosphere microbial community under fusarium wilt disease versus that in “healthy” soils. We found that variable selection process dominated in diseased rhizosphere bacterial communities, while stochastic processes dominated the assembly process within healthy sample microbiomes. This suggests the presence of a strong microbial selection pressure within the diseased plant rhizosphere. Recent advances in metabolomics have greatly advanced our understanding of plant-microbe interactions. Within the rhizosphere soil, plants exude organic metabolites to support microbial activity and, in turn, receive beneficial services from soil microbes [28]. A multistep model for root microbiome assembly from bulk soil has been proposed and supported with rice [29] and grapevines [30]. Dynamic root exudate profiles were associated with microbial community assembly patterns in a reference plant: wild oat (*Avena barbata*) [21]. These interactions appear to be two-way, as microbiomes were shown to condition soils by reprogramming root exudation profiles [31]. Specific root exudates have been associated with *F. oxysporum* disease spread in *Lisianthus* [20]. In this study, rhizosphere metabolites differed between diseased and healthy samples across multiple sites. Five metabolites (tocopherol acetate, citrulline, galactitol, octadecylglycerol, and behenic acid), enriched in the diseased rhizosphere soil, were considered to be “key” components that drove microbial community assembly. Several lines of evidence in literature indicate that these metabolites are associated with biotic stresses. Among these, citrulline has been found to be enriched in plants when exposed to multiple stressors [32] and also in the rhizosphere of fusarium wilt-diseased watermelon [33]. Tocopherol acetate is a member of the vitamin E family and is increased in host plants under multiple stresses [34]; meanwhile, behenic acid was enriched in sesame upon salinity stress [35]. We found higher abundances of tocopherol acetate and behenic acid in diseased rhizosphere soils, which appeared to be vital to the deterministic process of assembly within the diseased microbial community in our validation experiment. Thus, we suggest that the enrichment of some exudate constituents may be a common response of host plants to biotic and abiotic stresses. Nonetheless, not all rhizosphere metabolites alter microbial community assembly processes. For example, in a previous study [36], four organic acid exudates from cucumber (citric acid, pyruvate acid, succinic acid, and fumarate) were shown not to affect the microbial community assembly process (Supplementary Fig. 14). Collectively, the enrichment of five selected metabolites here within diseased rhizosphere soils may play an important role in the process of rhizosphere microbial community assembly as well as plant susceptibility to disease.

We found the ability of autotoxin degradation was decreased in diseased rhizosphere soil, which would be one of the mechanisms of disease happening as previous studies have shown that autotoxin accumulation would cause continuous cropping obstacles by nutrient imbalance and microbial dysfunction [7, 37, 38]. We further found that these functional abilities decline due to the decrease of relative abundance within *Bradyrhizobium*, *Streptomyces*, *Variovorax*, *Pseudomonas*, and *Sphingomonas*. These bacterial groups have been reported to play multiple beneficial roles, such as antibiotics production, root colonization, and ISR activation, to maintain plant health [3, 39–42]. Conversely, the metabolic of “readily available carbon” (small-molecule sugars and organic acids) was significantly enriched in diseased soil. These could promote the readily available carbon metabolism and, thus, increase the emergence and abundance of pathogens [43, 44]. Small-molecule organic acids could help plants defend against Fusarium wilt in several ways, such as pathogen growth inhibition [45], resistance improvement [45], and beneficial microorganisms recruitment [46]. However, the enhancement of organic acids metabolism leads to the weakness of the above potential beneficial function. Five feature microbes, *Anaeromyxobacter*, *Bdellovibrio*, *Conexibacter*, *Gemmatimonas*, and *Flavobacterium*, were the main contributors to small-molecule organic acids metabolism. Previously, all of the five bacterial groups have been uncovered in soil and/or rhizosphere environments, with one, *Conexibacter*, even being recognized as a pathogen [47]. Hence, both FM1 and FM2 may have important ecological roles in maintaining the health status of plants. However, further research is needed to verify the roles of these “potentially important” species in maintaining plant health or in the formation of the fusarium wilt-diseased microbial community.

## Conclusion

In this study, metabolites that were more abundant in diseased soils shaped the mechanisms by which microbial communities assemble and correspondingly the community compositions. The characteristics of the soils treated with these exudates were similar to natural diseased soils surveyed from many locations and cropping systems. This was consistent both in terms of the relative abundances of the distinguishing taxa and with regard to deterministic processes driving community assembly. Soils with these characteristics exhibited a higher disease incidence of fusarium wilt in watermelon. Together, our study revealed inherent differences in the composition of diseased and “healthy” rhizosphere microbiomes and identified dominant rhizosphere metabolites that drove the assembly of metabolite-responsive microbial groups contributing significantly to the characteristics of a diseased rhizosphere microbiome. This study provides a theoretical framework for the underlying causes in the establishment of a “diseased-state” rhizosphere microbial community that informs future control of fusarium wilt disease.

## Materials and methods

### Assessment of rhizosphere microbiome assembly process using sampling data

#### Rhizosphere soil collection from field

Rhizosphere soils of banana (B), cucumber (C), watermelon (W), and lily (L) were collected from Hainan, Guangdong, Beijing, and Jiangsu provinces, respectively (Supplementary Table 1). For diseased samples, plants with typical symptoms of fusarium wilt as necrotic, vascular, and root wilts were selected from plots cropped continuously for at least 3 years. Then, diseased root tissue was ground, diluted, and coated in on Nash-Snyder Fusarium-selective growth medium. The plates were incubated at 28 °C for 2 days, and the plate with distinct Fusarium colonies was visible and finally confirmed as diseased samples. Newly reclaimed plots without evidence of wilt disease were selected for the sampling of healthy rhizosphere soils. The healthy plots were generally selected proximal to the diseased plots in order to avoid biases due to geographical factors. Fifteen plants were obtained that represented healthy and diseased rhizosphere soils. Soils from three plants were pooled as one replicate, for a total of five replicates for each group. Finally, four pairs of diseased/healthy samples were obtained: BD/BH, diseased/healthy samples of banana; CD/CH, diseased/healthy samples of cucumber; WD/WH, diseased/healthy samples of watermelon; and LD/LH, diseased/healthy samples of lily. The rhizosphere soil was obtained as follows: soil loosely adhered to the plant roots was shaken off and discarded, and then the root tissues with their associated rhizosphere soil were cut into 1 cm segments by using a sterile scalpel under aseptic conditions. Soil tightly bound to the root segments was rinsed using sterile water. Half of the suspension from four pairs of samples was lyophilized for the rhizosphere metabolome analysis; another half of the suspension was centrifuged at 10,000 g for 10 min, and the pellet was collected for DNA extraction.

#### Rhizosphere soil collection from pot experiment

A continuous cropping pot experiment was conducted to mine the process from health to disease. The soil used in this experiment was collected with top soil (20 cm) from a field without a history of cucumber cultivation in Baimao town of Changshu city, China (31°35′36.19″N, 120°54′54.93″E). Soil chemical properties were as follows: pH 7.2, available P 21.60 mg/kg, available K 23.11 mg/kg, C/N 8.60, total K 1590.72 mg/kg, total N () 1.64 g/kg, total C 14.12 g/kg, and total P 0.54 g/kg. The collected soil was sieved (2-mm sieve) to remove plant debris and rocks after being air-dried, subsequently homogenized and stored at room temperature. The watermelon seeds were surface sterilized with 75% ethanol for 30 s and then 2% NaClO for 5 min before germination. Then, three seedlings were planted in each pot (length × width × height = 10 × 10 × 12 cm, containing 300 g soil) and randomly placed in a growth chamber (28/26 °C day-night, 70% relative humidity, 180 μmol light m^−2^ s^−1^). Plant tissues were removed from the soil 50 days after transplantation, and rhizosphere soil samples were harvested. Subsequently, the soils were placed back into the same pots for the next generation without cross mixing. The experiment was terminated at the eighth generation when serious Fusarium wilt symptoms occurred. Finally, original soil and the rhizosphere soils from the first, fifth, and eighth generations were used for microbiome analysis.

#### Rhizosphere microbiome analyses

Genomic DNA from 0.5 g soil was extracted with the PowerLyzer PowerSoil DNA Isolation Kit (Qiagen, Germany) following the manufacturer’s protocol. DNA quality and quantity were evaluated on a 1% agarose gel and with a NanoDrop 2000 spectrophotometer (Thermo Scientific, Waltham, MA, USA). For taxonomic profiling, PCR products that targeted the V4 region of the bacterial 16S rRNA gene were amplified with the primers 515F: GTGYCAGCMGCCGCGGTAA and 806R: GGACTACNVGGGTWTCTAAT) [48] to yield an amplicon of 292 bp. The 50 μL reaction mixtures contained 25 μL 2× Premix Taq (Takara Biotechnology, Dalian Co. Ltd., China), 1 μL each primer (10 μM), 3 μL DNA (20 ng/μL), and 20 μL of sterilized ultrapure water. PCR amplification was performed by using a Bio-Rad S1000 (Bio-Rad Laboratory, CA, USA) with the following cycles: 95 °C for 5 min, then 30 cycles of 94 °C for 30 s, 52 °C for 30 s, and 72 °C for 30 s with a final extension at 72 °C for 10 min. Products were run on a 1% agarose gel, and The DNA marker used was DNA Marker (100–2000 bp; B500350 Sangon Biotech (Shanghai) Co., Ltd.), and those with clear bands between 290 and 310 bp were combined for sequencing. PCR products were mixed at equal densities according to the GeneTools analysis software (version 4.03.05.0, SynGene), and the mixture was purified with an E. Z. N. A. Gel Extraction Kit (Omega, USA). Sequencing libraries were generated using the NEBNext^®^ Ultra™ DNA Library Prep Kit for Illumina^®^ (New England Biolabs, USA) following the manufacturer’s recommendations. Indexing barcodes were added, and the library quality was assessed with a Qubit^®^ 2.0 Fluorometer (Thermo Scientific) and an Agilent Bioanalyzer 2100. Finally, the library was sequenced on an Illumina Hiseq 2500 platform (Magigene, Guangdong). The 250-bp paired-end reads were filtered to obtain high-quality clean reads using Trimmomatic (V0.33, http://www.usadellab.org/cms/?page=trimmomatic), and sequences were assigned to each sample based on its unique barcode. For microbial community analysis, Bray-Curtis similarity matrices were prepared with the beta_diversity.py script. Principal coordinate analysis (PCoA) plots were generated from Bray-Curtis similarity matrices by using the R package “ggplot2” [49].

### Analysis of the microbial community assembly processes

We used two approaches to examine bacterial community phylogeny. First, the neutral model was applied [50] with the R code contributed by Burns et al. [51]. We estimated each OTU’s abundance in the metacommunity (pi) by averaging its relative abundance across all samples with the detection threshold (d) set to 1/N. The model was generated using the function *pbeta* from package “stats” (R Core Team 2018) and fit to data using function *nlsLM* from package “minpack.lm” [52]. The function *binconf* from the “Hmisc” package [53] was used to calculate a 95% prediction interval. Next, the null modeling approach was used to evaluate phylogenetic patterns of the rhizosphere microbiome by calculating the β-nearest taxon index (βNTI) between pairs of samples as described in Stegen et al. [54]. Before the βNTI calculation, we determined the observed weighted abundances of β-mean-nearest taxon distances (βMNTD) with the function *comdistnt* using the R package “picante” [55] and then generated the βMNTD null model by randomly shuffling the tips of the phylogenetic tree. The pairwise βMNTD values were recalculated 999 times to generate a null distribution for each pair. Then, the βNTI was calculated for each pairwise sample comparison among the entire metacommunity. Sample pairs with |βNTI| > 2 are expected to result from deterministic processes [54], while |βNTI| < 2 values indicate that selection pressure is weak and community assembly is likely governed by stochastic processes [55]. Values derived from βNTI analyses reflect the driving force of factors that influence community assembly processes as phylogenetic turnover correlates with environmental dissimilarity. Mantel tests (package “vegan” [56]) were performed to evaluate whether βNTI values were significantly different.

In order to examine the role of dispersal in the process of community assembly, we examined OTU turnover using the weighted abundance based on the Raup-Crick metric (RCbray), as reported by Stegen et al. [54]. RCbray determines whether OTU turnover between samples deviates from the expectations of ecological drift alone. We first determined the Bray-Curtis dissimilarity between each pair of samples. For each sample, we then randomly generated a null-model community with the same size and richness. Each null model was constructed by selecting OTUs randomly (weighted by frequency across all samples) with their relative abundance determined by their relative abundance in the metacommunity. We then determined the Bray-Curtis dissimilarity between all pairs of simulated communities. This process was repeated 999 times to generate a null-model distribution. RCbray was calculated by adding the number of simulated communities with a Bray-Curtis dissimilarity greater than the observed dissimilarity (Nsim > obs) to one-half of the number of simulated communities with a Bray-Curtis dissimilarity equal to the observed dissimilarity (Nsim = obs) and dividing this by the total number of simulations (999). Sample pairs with a |βNTI| < 2 and an |RCbray| > 0.95 indicated that bacterial community turnover was dominated by dispersal. More specifically, limited dispersal was determined to be dominant when RCbray > 0.95, while RCbray < −0.95 indicates homogenizing dispersal [54]. We combined the results from the βNTI and RCbray analyses to determine the relative proportion of the overall community assembly governed by deterministic and stochastic processes within each sample.

### Assessment of rhizosphere microbiome assembly process using integrated sequence data

#### Data collection and description

Bacterial/archaeal 16S rRNA sequencing results and metadata related to fusarium wilt disease in healthy and diseased rhizosphere samples were collected from 45 studies with 1722 samples (Supplementary Table 2) by searching the keywords “Fusarium wilt microbiome,” “Fusarium wilt community,” and “Fusarium wilt structure” in Google Scholar and the NCBI SRA database. These datasets included samples from the rhizosphere soils of healthy plants without symptoms of fusarium wilt and diseased samples collected from the rhizospheres of plants with symptoms of fusarium wilt. Sequencing data were generated from the Roche 454 (11.1%) and Illumina sequencing platforms (88.9%) (Supplementary Table 2). In total, eleven different primer pairs (515F:806R; 515F:907R; 338F:806R; 520F:802R; 799F:1193R; 27F:533R; 563F:802R; 341F:785R; 341F:805R; 27F:533R; and 27F:518R) were used that accounted for 82% of the samples, with the majority (55.6%) reflecting amplification from the V4 or V3-V4 regions of the 16S rRNA gene (Supplementary Table 2; Supplementary Fig. 1).

### Exploring microbial features and characterizing phylogenic patterns

The processing procedure for the sequencing data was detailed in our previous publication [13]. Briefly, the high quality of all of the sequencing reads was verified using FastQC v.0.11.5 [57], and paired-end reads were merged and then trimmed with usearch [58]. Due to the different amplified regions of the 16S rRNA gene among the collected sequencing data, all sequencing data from multiple studies were clustered using unoise3 in usearch [58], respectively. Species annotation was performed on the OTUs representative sequence from each study through the SSUrRNA database of SILVA (version: 138; http://www.arb-silva.de/). Then, the OTUs representative sequence was aligned to the greengene database (version: 13.8), and the best match sequencing ID was used for building an evolutionary tree by filtering subtree from the rep_set_99.tree in the greengenes database.

To address PCR biases, OTU filtration was performed with two strategies based on our previous publication [13]. Relative abundance was used to standardize the OTU profiles by scale_micro script in R package “ggCLusterNet.” For *alpha* diversity analysis, the OTUs were rarefied to 2000 reads per sample, and Chao1, Shannon, and Pielou_evenness indices were calculated in R using the “vegan” package. Bray-Curtis dissimilarity matrices were prepared with the beta_diversity.py script (Qiime-1.9.1) for *beta* diversity calculation and ordination (principal component analysis, principal coordinate analysis, nonmetric multidimensional scaling) plots were generated from Bray-Curtis dissimilarity matrices by using the R package “ggplot2.” Cluster analysis, based on ordination data sets, was performed with the cluster R package, and ellipses for each cluster were added with the “ggplot” package. Significant correlations between the relative abundances of bacterial OTUs were calculated using the sparse correlations for compositional data algorithm implemented in the R package “SpiecEasi” and plotted using the R package “ggClusterNet” [59]. Only the absolute values of correlation coefficient (“R-corr”) were greater than 0.6, and *p*-values less than 0.05 were plotted. In order to describe the topology of the resulting network, a set of measures (average node connectivity, average path length, diameter, cumulative degree distribution, clustering coefficient, and modularity) were calculated [60]. All statistical analyses were carried out in the R environment (http://www.r-project.org) using the “vegan” [60] and “igraph” packages [59]. To assess nonrandom patterns in the resulting network, we compared our network against its randomized version using the “igraph” package. Structural attributes of this network, such as the clustering coefficient and characteristic path length, were compared with those in the random network with equal nodes and edges. Analyses of microbial community assembly followed the protocol as described above.

### Rhizosphere metabolome detection

To identify rhizosphere metabolites that could drive microbiome assembly in the rhizosphere of diseased plants, rhizosphere metabolites were extracted with four pairs of samples (BD/BH, CD/CH, LD/LH, WD/WH) and analyzed according to our previous method with some modifications [61]. Briefly, rhizosphere soils were extracted twice with methanol solution (*V*_methanol_: *V*_H2O_ = 3:1) and ethyl acetate. The extractions were combined for drying by adding 20 μL methoxyamination hydrochloride, followed by incubation for 30 min at 80 °C before being treated with 30 μL of BSTFA (bis (trimethylsilyl) trifluoroacetamide) reagent (1% trimethylchlorosilane, v/v). The mixture was then incubated for 1.5 h at 70 °C and finally analyzed with a gas chromatograph (Agilent 7890) coupled with a GC-TOF-MS (Shanghai Biotree Biotech Co. Ltd.). Raw peak analyses were performed as reported by Wen et al. [36].

For the differences among groups, relative abundances were used to standardize the metabolite profiles, and Bray-Curtis similarity matrices were prepared using the R package “vegan.” Permutational multivariate analysis of variance (PERMANOVA; Adonis, transformed data by Bray-Curtis, permutations = 999) was used to determine significant differences in *beta* diversity, and principal coordinate analysis (PCA) plots were generated from Bray-Curtis similarity matrices using “ggplot2” in R. Network analyses were performed using the R package “ggClusterNet” [62].

In order to determine metabolites that may drive the process of microbial community assembly in the diseased rhizosphere, machine learning was used to distinguish the rhizosphere metabolites associated with diseased and healthy rhizosphere soils. Because we found lower model accuracies when the models were built with all of the detected rhizosphere metabolites, we then trained a series of random forest models based on cutoff values for enriched metabolites characterized by relative abundances ranging from 1 to 90% and found the greatest accuracy in those trained with metabolites at > 3% (Supplementary Table 4). To avoid omitting important metabolites, we also trained a series of models from low-abundance metabolites (< 3%) and found that the greatest accuracy occurred with metabolite abundances at < 1% (Supplementary Table 4). The “important” metabolites were selected by cross-tabulations in R with “randomForest.” Wilcoxon tests (“stat” package) were conducted in order to detect the differences in rhizosphere metabolites between the diseased and healthy samples. Metabolites deemed as “important” from the classifiers and those that were significantly different between the two groups were selected for correlation analysis (the maximum-entropy approach) with their relative abundance and βNTI value in the diseased samples. Those metabolites significantly associated with the process of microbial community assembly were selected for further confirmation.

### Effects of specific metabolites on the soil microbial assembly process and fusarium wilt disease occurrence

#### Soil condition experiment

To evaluate the effects of specific rhizosphere metabolites on the soil microbial assembly process and fusarium wilt disease occurrence, two soils were collected from fields without a history of fusarium wilt disease occurrence (one from Lvliang, Shanxi province, recorded as S1. The other from Yulin, Shanxi province, recorded as S2) were used for incubations with the potentially active metabolites selected above (tocopherol acetate, citrulline, galactitol, octadecylglycerol, and behenic acid). Physicochemical properties of soils are detailed in Supplementary Table 3. Prior to the incubations, 50 g of soil was introduced into 9-cm-diameter Petri dishes and incubated in a growth chamber at 28 °C, 2 weeks for soil microbiome equilibration. Indigenous seeds were germinated by irrigation with 5 mL autoclaved water twice a week and removed. Following the incubation, 5 mL of solution containing the selected metabolites was added to each plate twice a week for 8 weeks.

Two solutions containing equal proportions of tocopherol acetate, citrulline, galactitol, octadecylglycerol, and behenic acid were prepared at final total concentrations of 1 μM (C1 solution) and 100 μM (C2 solution). The C1 mixture contained 0.2 μM each of tocopherol acetate, citrulline, galactitol, octadecylglycerol, and behenic acid, whereas the C2 mixture contained 20 μM each of those same metabolites. For each soil, there were two treatments and one control that were amended with autoclaved water (S1C1, S1C2, and S1CK; S2C1, S2C2, and S2CK). Each treatment contained ten plates, and all plates were randomly arranged during the incubation. Soil moisture was maintained by determining the mass twice a week and adjusting to 70% of water holding capacity using sterile deionized water. After 8 weeks, soils from all ten plates of each treatment were collected. Per plate, 45 g of soil was stored at 4 °C and used for the soil culture experiment. Subsamples of 5 g soil were randomly selected from six of the ten replicate plates and stored at −80 °C for 16S rRNA sequencing. The process of raw sequencing data, diversity estimations, and determination of the microbial community assembly was identical to the aforementioned process.

### Impacts of metabolites on fusarium wilt disease incidence

Fifty pots (3.5 × 3.5 × 5 cm), each containing 10 g fresh steam-sterilized vermiculite, were established for each treatment and divided into 5 replicates. Next, 1 g of soils that were conditioned with the selected metabolites was suspended in 9 mL sterile water, filtered through sterile Whatman 42 filter paper to remove the large particles of soil, and then irrigated to each pot. Growth chamber conditions for seed germination and the potted experiment were as follows: 16 h light at 25 °C, 46% relative humidity and 8 h darkness, and 18 °C at 37% relative humidity. Watermelon seeds “8424” (Xinjiang Farmer Seed Technology Co., Ltd.) were sterilized with NaClO solution (0.75%, v/v) for 30 min and sown on MS medium supplemented with 1% sucrose for 5 days before being transferred to the pots. After 7 days of watermelon seedling growth, 5 mL of *Fusarium oxysporum* spores at a concentration of 2 × 10^5^ CFU mL^−1^ was irrigated into each pot. Autoclaved water and MS medium solution were added to maintain the growth of watermelon, and fusarium wilt disease incidence was monitored during the course of the experiment for 20 days based on the reported strategy [63]. Disease incidence was expressed as a percentage of diseased plants per the total number of plants and was based on observations of typical wilt symptoms [64].

### Metagenomic sequencing and data analysis

Samples from the soil condition experiment (S1C1; S1C2; S1CK; S2C1; S2C2; S2CK) were used for metagenomic sequencing. All the samples were subjected to shotgun metagenomic sequencing by using an Illumina HiSeq 2500 (2 × 150) instrument. The paired-end metagenomics shotgun sequencing data were then trimmed of adaptors, and low-quality (length less than 50 bp, with a quality score less than 20, had N bases) paired-end reads were filtered to remove with KneadData (https://huttenhower.sph.harvard.edu/kneaddata/). The assembly of metagenomics data was performed by MEGAHIT [65]. Contigs over 300 bp were used for further gene prediction and annotation. Open reading frames (ORFs) from assembled metagenomes were predicted using MetaGeneMark. The predicted ORFs with lengths longer than 100 bp were retrieved and translated into amino acid sequences through the NCBI translation table. A nonredundant gene catalog was constructed using CD-HIT [66] with criteria of 95% sequence identity combined with 90% coverage, and then gene abundance in each sample was evaluated. For taxonomic annotation, the representative sequences of the nonredundant gene catalog were searched against the nonredundant protein database of NCBI with an *e*-value cutoff of 1e-5 using DIAMOND. Lowest common ancestor method was applied to estimate the assignment of genes to specific taxa. Annotation of the functional genes was performed using the “emapper.py” function in EggNOG [67]. The count number of KEGG annotation was filtered for downstream comparison. Gene abundances were normalized into reads per kilobase million counts. The GSVA analysis was performed using R package GSVA [68]. The bubble plots were generated using the R package ggplot2. Heatmap of functional pathways and relative taxonomic abundances were generated by ggplot2.

## Supplementary Information

**Additional file 1:** **Supplementary Figure 1.** Merging of sequencing metadata from independent studies. Country and zones (A), amplicon region (B), primers (C) and sequencing platforms (D) used in this study are displayed. **Supplementary Figure 2.** Principal coordinates analysis (PCoA) with Bray-Curtis dissimilarity performed on rhizosphere metabolites. R- and *P*-values were evaluated via Adonis test. BD: diseased banana, BH: healthy banana (from Hainan); CD: diseased cucumber, CH healthy cucumber (from Guangdong); WD: diseased watermelon, WH: healthy watermelon (from Beijing); LD: diseased lily, LH: healthy lily (from Hunan). **Supplementary Figure 3.** The accuracy of Random Forest models built with microbes belonging to the five genera in our sequencing data. **Supplementary Figure 4.** The accuracy of Random Forest models built with microbes belonging to the five genera in the integrated metadata. Supplementary Figure 5. Principal coordinates analysis (PCoA) with Bray-Curtis dissimilarity performed on the taxonomic profile (at the OTU level) of compounds in conditioned soils. R- and *P*-values were evaluated via Adonis test. **Supplementary Figure 6.** Principal coordinates analysis (PCoA) with Bray-Curtis dissimilarity performed on the metagenome profile of two soils conditioned with metabolites. S1C1 means soil1 conditioned by metabolites at concentration of 1 μM; S1C2 means soil1 conditioned by metabolites at concentration of 100 μM; S2C1 means soil2 conditioned by metabolites at concentration of 1 μM; S2C2 means soil2 conditioned by metabolites at concentration of 100 μM. **Supplementary Figure 7.** GSVA was performed to identify significantly enriched (*P*-value < 0.05, two-sided unpaired limma) biological pathways between S1C1 and S1CK. Bubbles indicated GSVA enrichment score of these pathways. **Supplementary Figure 8.** GSVA was performed to identify significantly enriched (*P*-value < 0.05, two-sided unpaired limma) biological pathways between S1C2 and S1CK. Bubbles indicated GSVA enrichment score of these pathways. **Supplementary Figure 9.** GSVA was performed to identify significantly enriched (*P*-value < 0.05, two-sided unpaired limma) biological pathways between S2C1 and S2CK. Bubbles indicated GSVA enrichment score of these pathways. **Supplementary Figure 10.** GSVA was performed to identify significantly enriched (*P*-value < 0.05, two-sided unpaired limma) biological pathways between S2C2 and S2CK. Bubbles indicated GSVA enrichment score of these pathways.  **Supplementary Figure 11.** Principal coordinates analysis (PCoA) with Bray-Curtis dissimilarity performed on the metagenome profile of rhizosphere soil samples collected from the 1st and 8th generation. **Supplementary Figure 12.** GSVA was performed to identify significantly enriched (*P*-value < 0.05, two-sided unpaired limma) biological pathways between 1st and 8th. Bubbles indicated GSVA enrichment score of these pathways. **Supplementary Figure 13.** The relative abundance information for FM1 and FM2 between 1st and 8th. **Supplementary Figure 14.** Contributions of deterministic and stochastic processes on community assembly in the soil treated with SMOAs and Control.

**Additional file 2:** **Supplementary Table 1.** Sampling information for diseased and healthy rhizosphere soils. **Supplementary Table 2.** Basic sequencing data for other reports integrated into our study. **Supplementary Table 3.** Soil chemical properties of the two soils used for the conditioned soil experiment. **Supplementary Table 4.** Selected different threshold (thresholda/b: represent selected the metabolites with the relative abundance greater/less than threshold for model 1/model2 construction) for model construction. **Supplementary Table 5.** Grouping of metabolites according to their chemical properties. **Supplementary Table 6.** Pairwise Adonis for multiple pairwise comparisons of rhizosphere metabolites. **Supplementary Table 7.** Total of 46 metabolites were significant difference and also in the best biomarkers identified by the random forest classifier between the two groups.

## Data Availability

The authors declare that the data supporting the findings of this study are available within the paper and its supplementary information files. Raw sequence data obtained in this study have been deposited in the Genome Sequence Archive in the BIG Data Center, Chinese Academy of Sciences, under accession code CRA004764. All scripts for computational analysis and corresponding raw data are available at https://github.com/taowenmicro/2022.Wentao.etal_fu-wilt-rhi-micro-assembly.
