# Supplementary material for: Specific metabolites drive the deterministic assembly of diseased rhizosphere microbiome through weakening microbial degradation of autotoxin
Source: Microbiome. 2022 Oct 21;10:177. doi: 10.1186/s40168-022-01375-z (PMC9587672; doi:10.1186/s40168-022-01375-z)

**Supplementary figures and tables for**

“Specific metabolites drive the deterministic assembly of diseased rhizosphere microbiome through weakening microbial degradation of autotoxin”

Supplementary Figure 1: Merging of sequencing metadata from independent studies. Country and zones (A), amplicon region (B), primers (C) and sequencing platforms (D) used in this study are displayed.


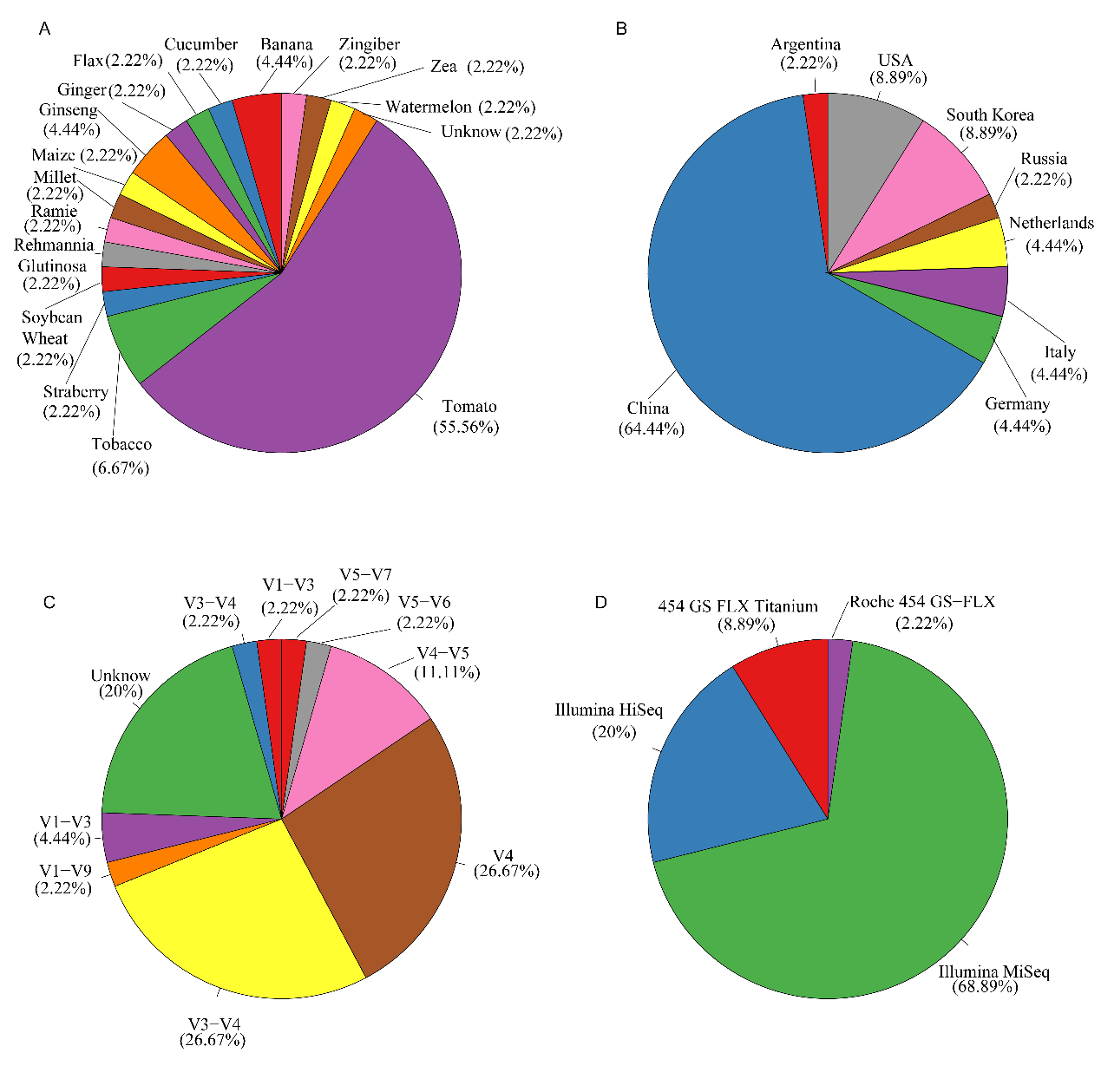


Supplementary Figure 2: Principal coordinates analysis (PCoA) with Bray-Curtis dissimilarity performed on rhizosphere metabolites. R- and P-values were evaluated via Adonis test. BD: diseased banana, BH: healthy banana (from Hainan); CD: diseased cucumber, CH healthy cucumber (from Guangdong); WD: diseased watermelon, WH: healthy watermelon (from Beijing); LD: diseased lily, LH: healthy lily (from Hunan).


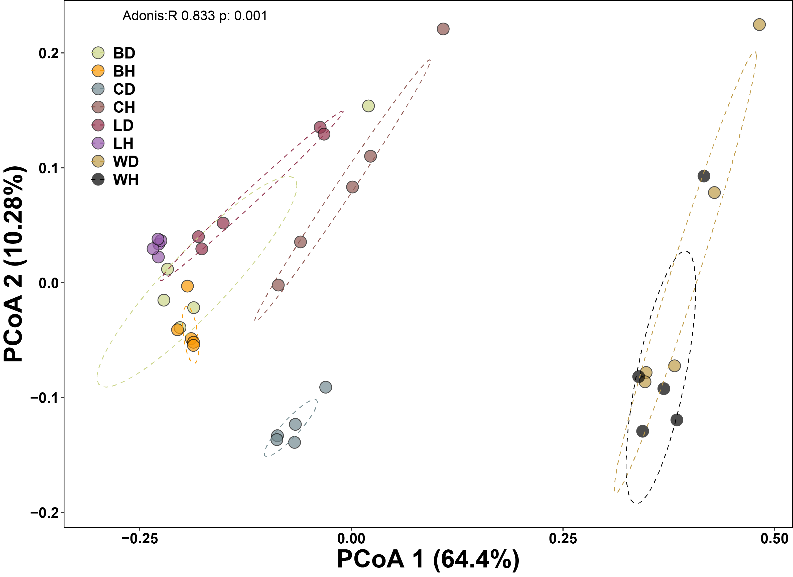


Supplementary Figure 3: The accuracy of Random Forest models built with microbes belonging to the five genera in our sequencing data


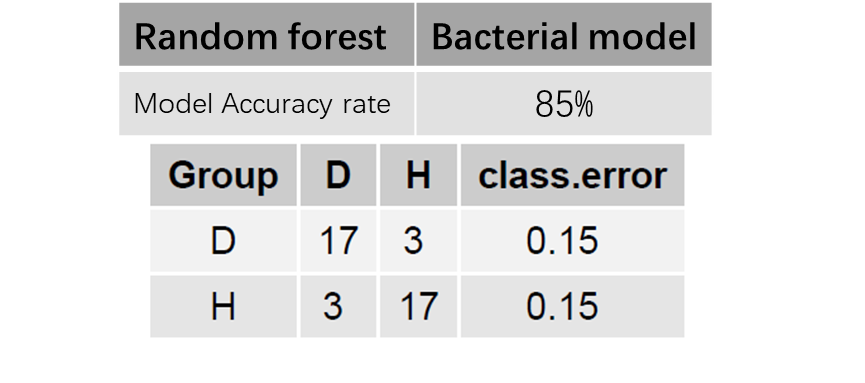


Supplementary Figure 4: The accuracy of Random Forest models built with microbes belonging to the five genera in the integrated metadata.


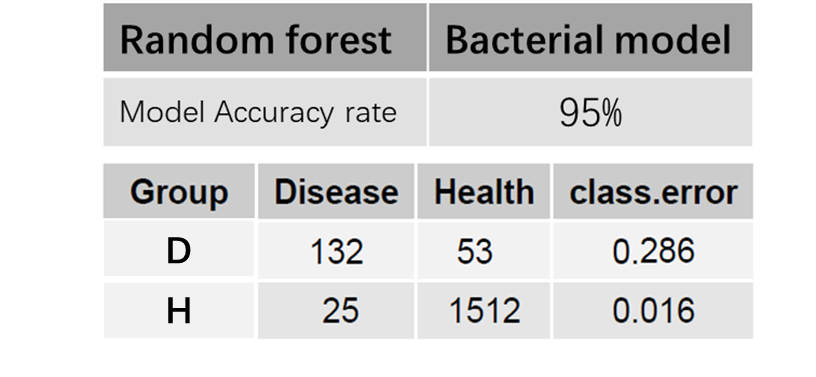


Supplementary Figure 5: Principal coordinates analysis (PCoA) with Bray-Curtis dissimilarity performed on the taxonomic profile (at the OTU level) of compounds in conditioned soils. R- and P-values were evaluated via Adonis test.


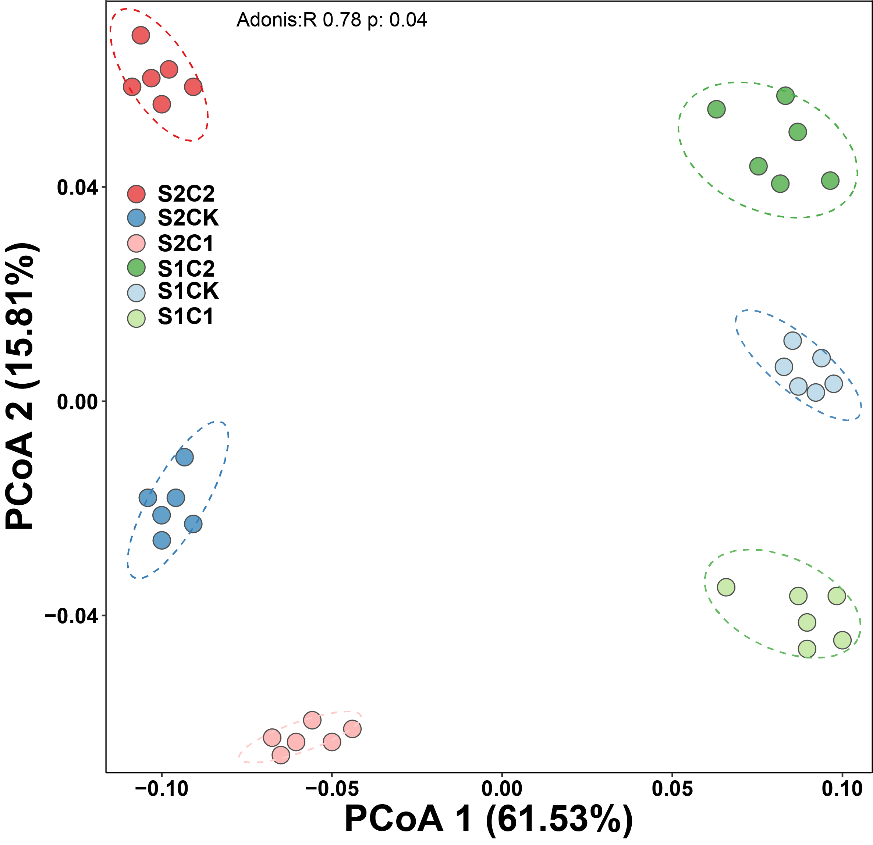


Supplementary Figure 6 Principal coordinates analysis (PCoA) with Bray-Curtis dissimilarity performed on the metagenome profile of two soils conditioned with metabolites. S1C1 means soil1 conditioned by metabolites at concentration of 1 μM; S1C2 means soil1 conditioned by metabolites at concentration of 100 μM; S2C1 means soil2 conditioned by metabolites at concentration of 1 μM; S2C2 means soil2 conditioned by metabolites at concentration of 100 μM.


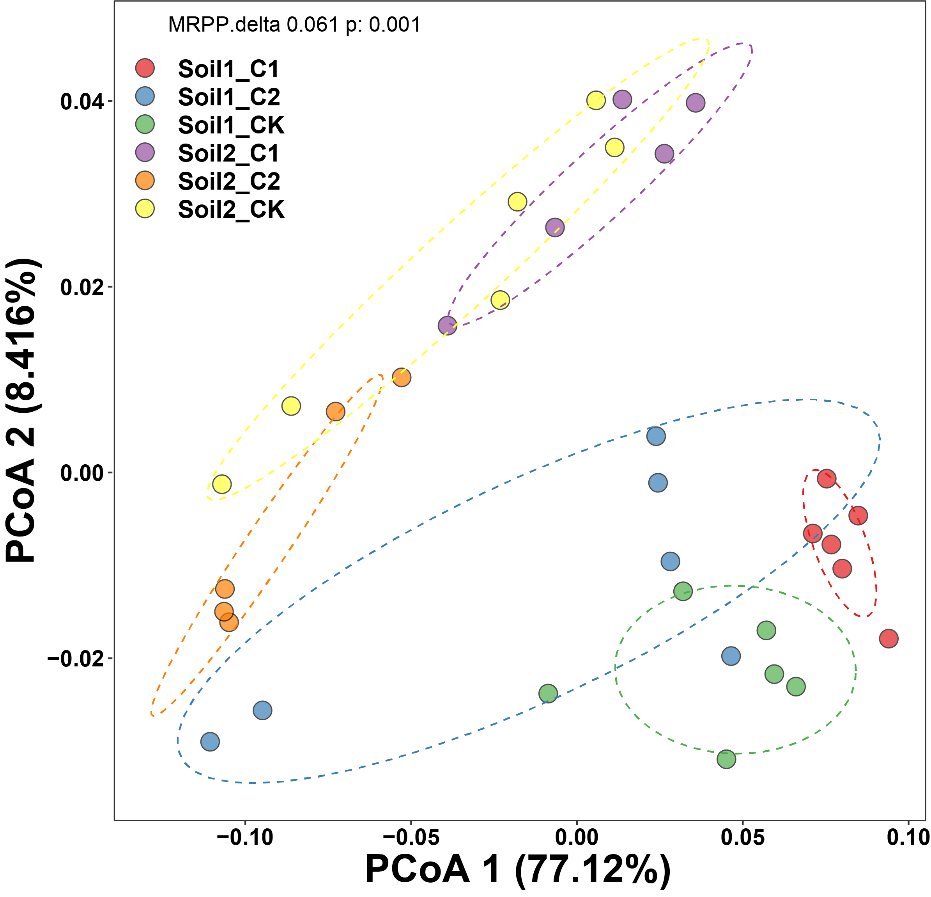


Supplementary Figure 7: GSVA was performed to identify significantly enriched (P-value < 0.05, two-sided unpaired limma) biological pathways between S1C1 and S1CK. Bubbles indicated GSVA enrichment score of these pathways.


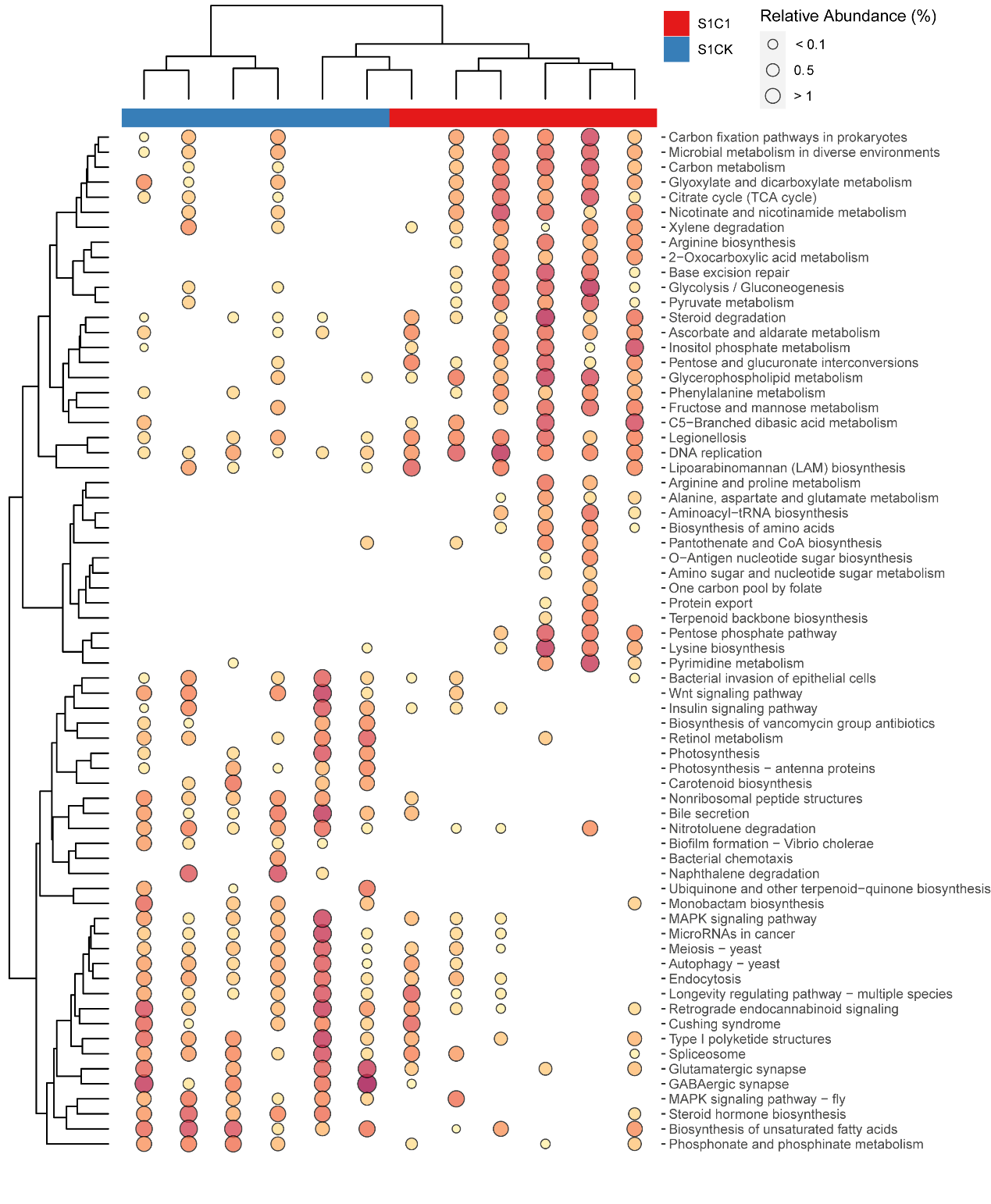


Supplementary Figure 8: GSVA was performed to identify significantly enriched (P-value < 0.05, two-sided unpaired limma) biological pathways between S1C2 and S1CK. Bubbles indicated GSVA enrichment score of these pathways.


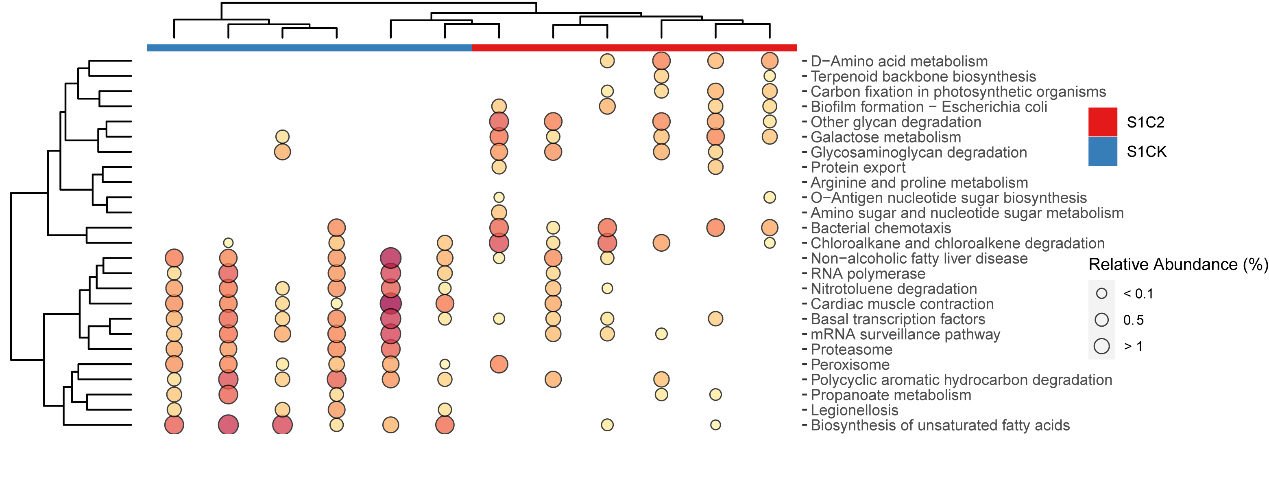


Supplementary Figure 9: GSVA was performed to identify significantly enriched (P-value < 0.05, two-sided unpaired limma) biological pathways between S2C1 and S2CK. Bubbles indicated GSVA enrichment score of these pathways.


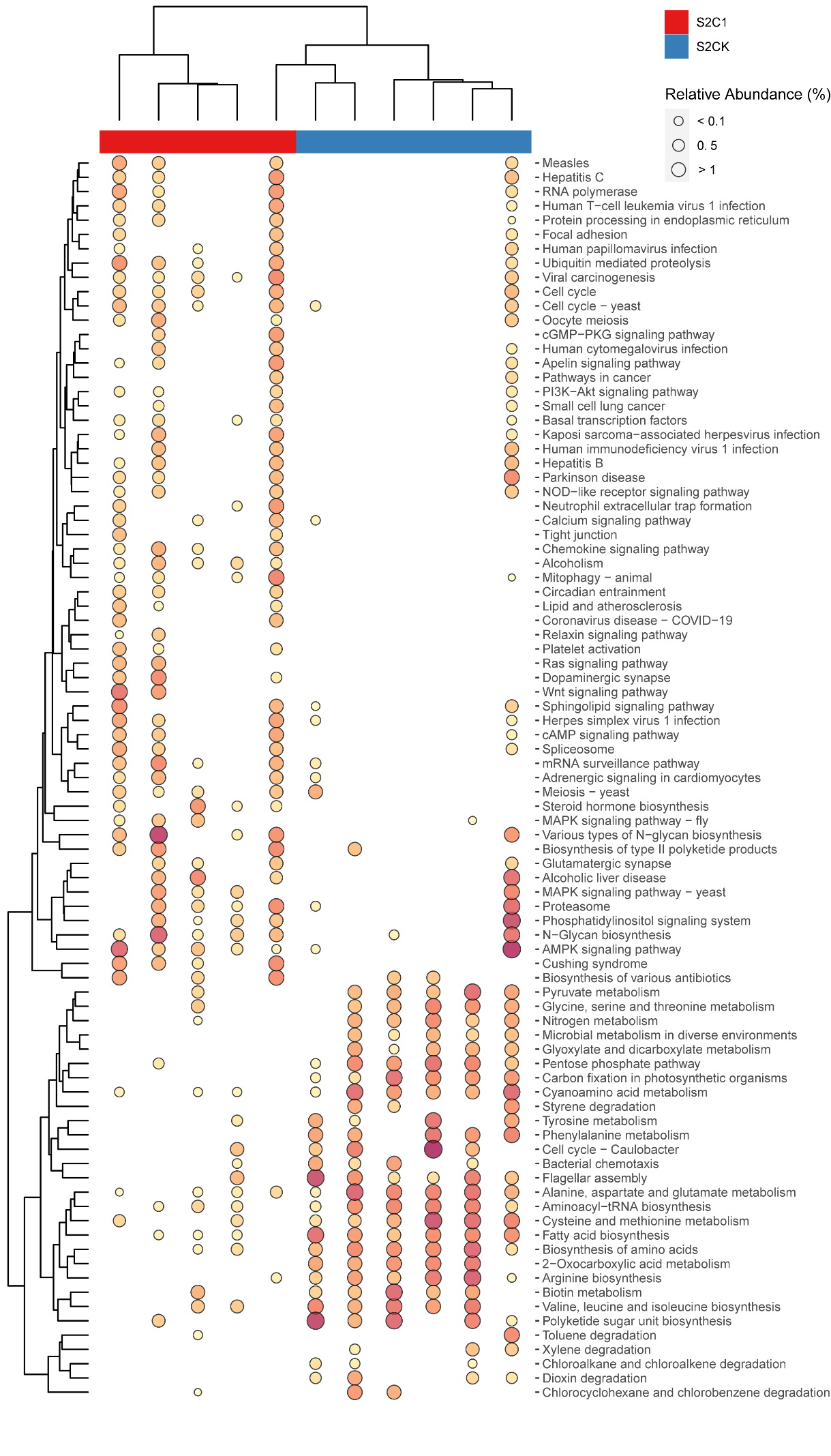


Supplementary Figure 10: GSVA was performed to identify significantly enriched (P-value < 0.05, two-sided unpaired limma) biological pathways between S2C2 and S2CK. Bubbles indicated GSVA enrichment score of these pathways.


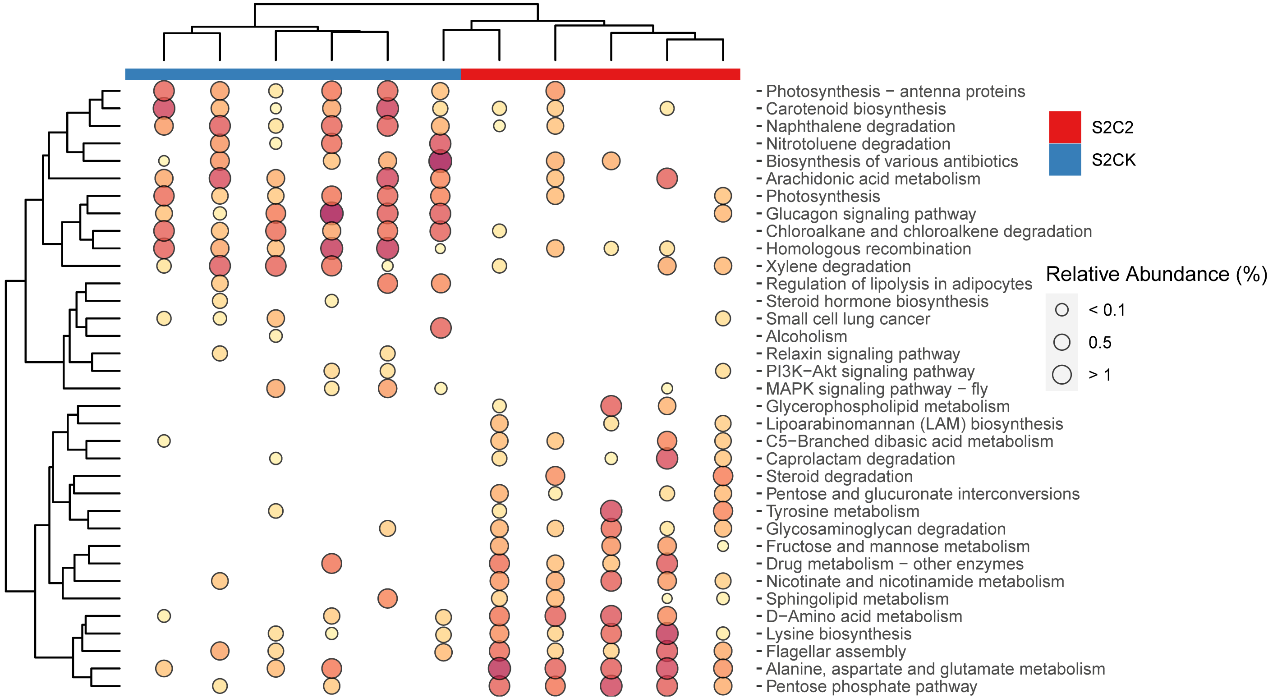


Supplementary Figure 11: Principal coordinates analysis (PCoA) with Bray-Curtis dissimilarity performed on the metagenome profile of rhizosphere soil samples collected from the 1^st^ and 8^th^ generation.


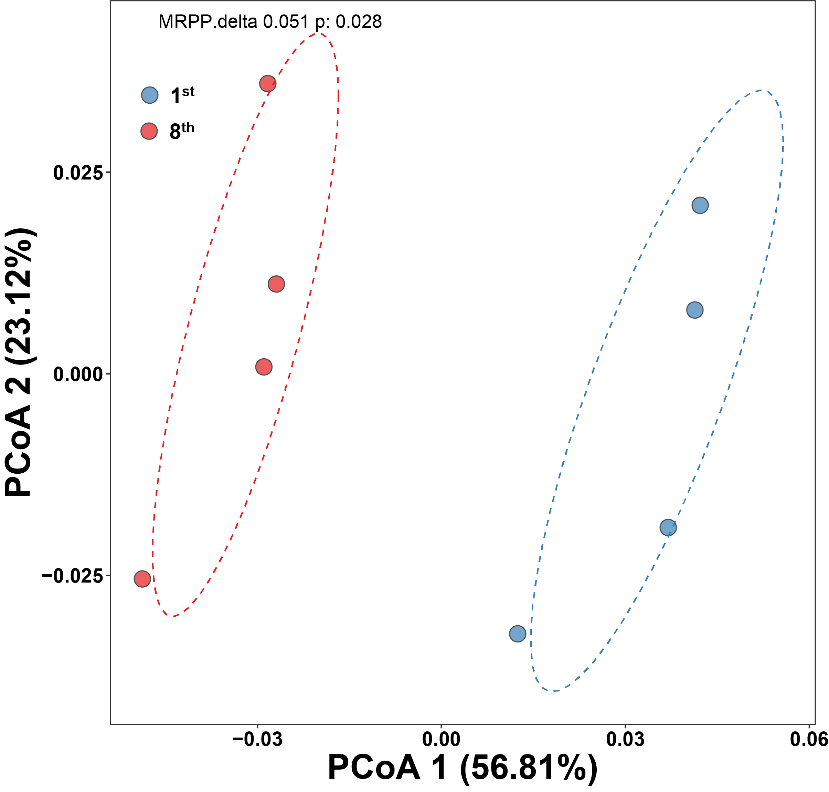


Supplementary Figure 12: GSVA was performed to identify significantly enriched (P-value < 0.05, two-sided unpaired limma) biological pathways between 1^st^ and 8^th^. Bubbles indicated GSVA enrichment score of these pathways.


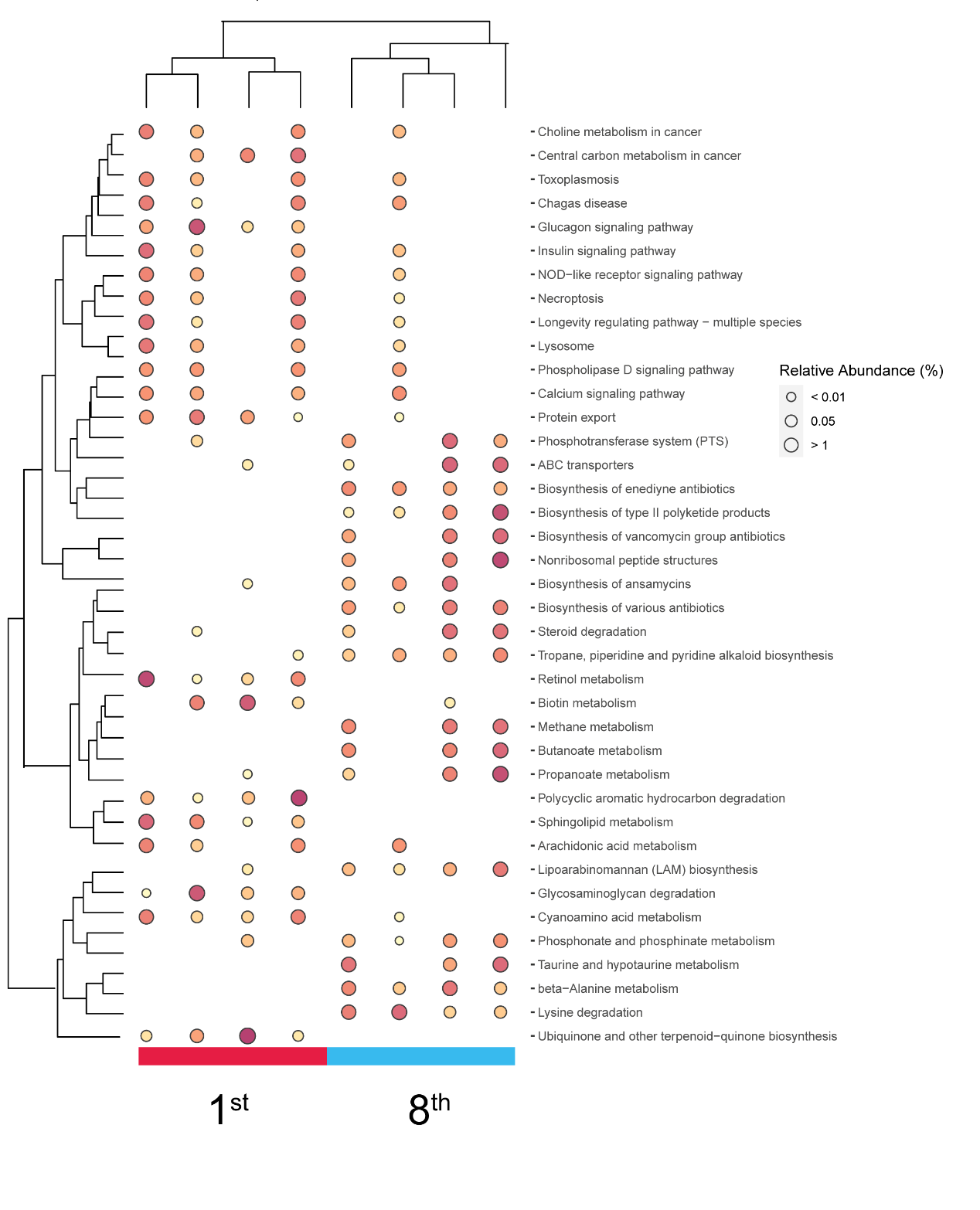


Supplementary Figure 13: The relative abundance information for FM1 and FM2 between 1^st^ and 8^th^.


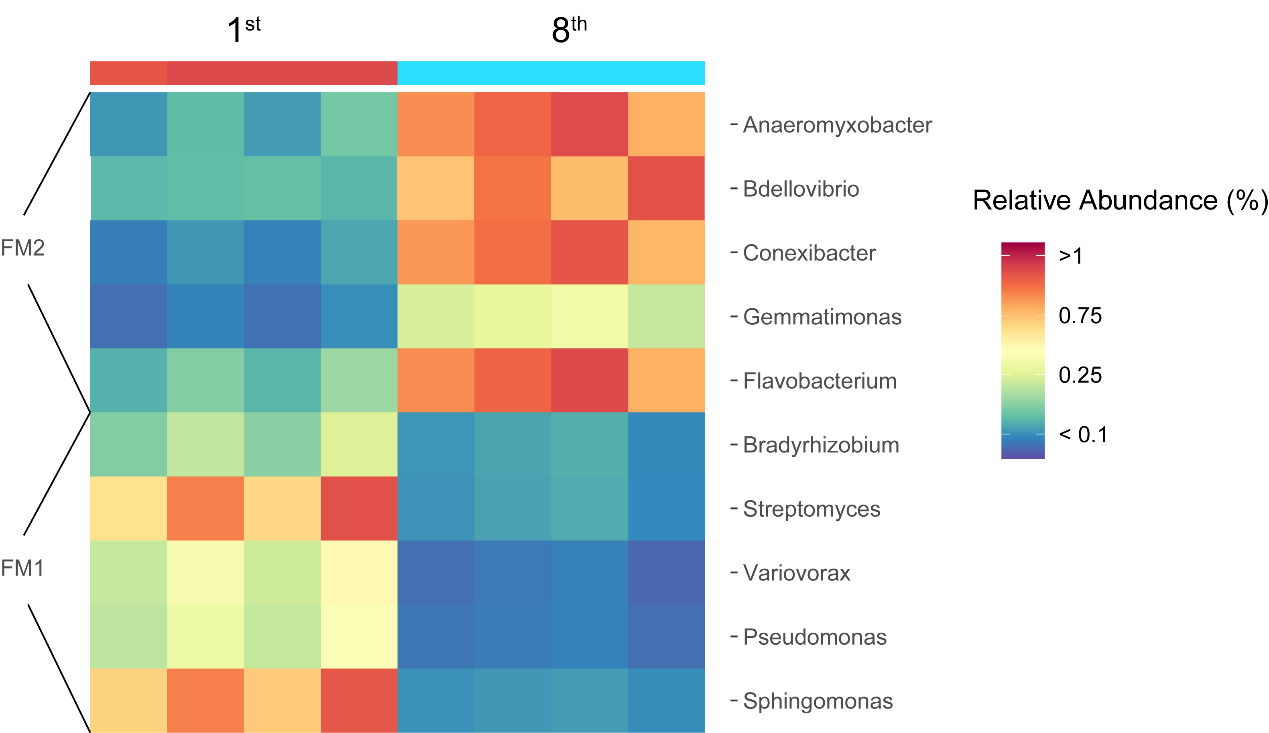


Supplementary Figure 14 Contributions of deterministic and stochastic processes on community assembly in the soil treated with SMOAs and Control.


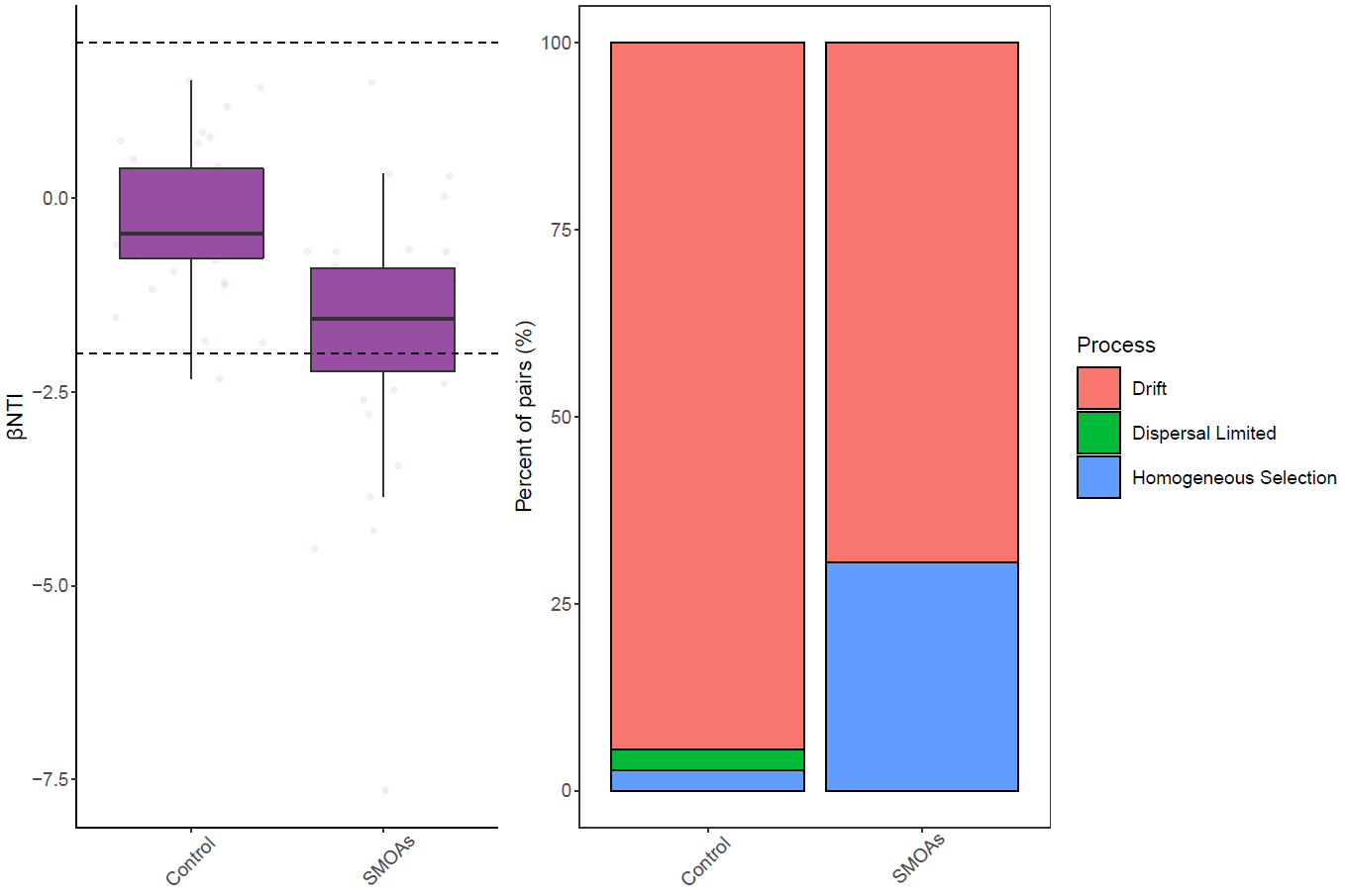

Supplement: Supplementary file 2 — Additional file 1: Supplementary Figure 1. Merging of sequencing metadata from independent studies. Country and zones (A), amplicon region (B), primers (C) and sequencing platforms (D) used in this study are displayed. Supplementary Figure 2. Principal coordinates analysis (PCoA) with Bray-Curtis dissimilarity performed on rhizosphere metabolites. R- and P-values were evaluated via Adonis test. BD: diseased banana, BH: healthy banana (from Hainan); CD: diseased cucumber, CH healthy cucumber (from Guangdong); WD: diseased watermelon, WH: healthy watermelon (from Beijing); LD: diseased lily, LH: healthy lily (from Hunan). Supplementary Figure 3. The accuracy of Random Forest models built with microbes belonging to the five genera in our sequencing data. Supplementary Figure 4. The accuracy of Random Forest models built with microbes belonging to the five genera in the integrated metadata. Supplementary Figure 5. Principal coordinates analysis (PCoA) with Bray-Curtis dissimilarity performed on the taxonomic profile (at the OTU level) of compounds in conditioned soils. R- and P-values were evaluated via Adonis test. Supplementary Figure 6. Principal coordinates analysis (PCoA) with Bray-Curtis dissimilarity performed on the metagenome profile of two soils conditioned with metabolites. S1C1 means soil1 conditioned by metabolites at concentration of 1 μM; S1C2 means soil1 conditioned by metabolites at concentration of 100 μM; S2C1 means soil2 conditioned by metabolites at concentration of 1 μM; S2C2 means soil2 conditioned by metabolites at concentration of 100 μM. Supplementary Figure 7. GSVA was performed to identify significantly enriched (P-value < 0.05, two-sided unpaired limma) biological pathways between S1C1 and S1CK. Bubbles indicated GSVA enrichment score of these pathways. Supplementary Figure 8. GSVA was performed to identify significantly enriched (P-value < 0.05, two-sided unpaired limma) biological pathways between S1C2 and S1CK. Bubbles indicated GSVA enri [file 40168_2022_1375_MOESM1_ESM.docx]
